# The *Plasmodium falciparum* Malaria M1 Alanyl Aminopeptidase (*Pf*A-M1): Insights of Catalytic Mechanism and Function from MD Simulations

**DOI:** 10.1371/journal.pone.0028589

**Published:** 2011-12-21

**Authors:** Peter M. Jones, Mark W. Robinson, John P. Dalton, Anthony M. George

**Affiliations:** 1 School of Medical and Molecular Biosciences, Sydney, New South Wales, Australia; 2 i3 Institute, University of Technology Sydney, Sydney, New South Wales, Australia; 3 Institute of Parasitology, McGill University, Montreal, Quebec, Canada; University of Cambridge, United Kingdom

## Abstract

Malaria caused by several species of *Plasmodium* is major parasitic disease of humans, causing 1–3 million deaths worldwide annually. The widespread resistance of the human parasite to current drug therapies is of major concern making the identification of new drug targets urgent. While the parasite grows and multiplies inside the host erythrocyte it degrades the host cell hemoglobin and utilizes the released amino acids to synthesize its own proteins. The *P. falciparum* malarial M1 alanyl-aminopeptidase (*Pf*A-M1) is an enzyme involved in the terminal stages of hemoglobin digestion and the generation of an amino acid pool within the parasite. The enzyme has been validated as a potential drug target since inhibitors of the enzyme block parasite growth *in vitro* and *in vivo*. In order to gain further understanding of this enzyme, molecular dynamics simulations using data from a recent crystal structure of *Pf*A-M1 were performed. The results elucidate the pentahedral coordination of the catalytic Zn in these metallo-proteases and provide new insights into the roles of this cation and important active site residues in ligand binding and in the hydrolysis of the peptide bond. Based on the data, we propose a two-step catalytic mechanism, in which the conformation of the active site is altered between the Michaelis complex and the transition state. In addition, the simulations identify global changes in the protein in which conformational transitions in the catalytic domain are transmitted at the opening of the N-terminal 8 Å-long channel and at the opening of the 30 Å-long C-terminal internal chamber that facilitates entry of peptides to the active site and exit of released amino acids. The possible implications of these global changes with regard to enzyme function are discussed.

## Introduction

Malaria is a major cause of human morbidity and mortality worldwide with an estimated 1–3 million deaths each year (http://www.who.int/malaria/wmr2008/malaria2008). Four Plasmodium species commonly infect humans, but *P. falciparum* is responsible for most deaths [1]. With the declining efficacy of affordable antimalarial drugs such as sulfadoxine-pyrimethamine due to the emergence of drug resistant parasites, the current recommended treatment for uncomplicated malaria in most countries relies on an artemisinin combination therapy (ACT). Recent reports suggesting a decreased efficacy of these ACTs [2], [3], [4] is of major concern and highlights the urgent need for next generation antimalarial agents that target new parasite systems.

Most anti-malarial drugs target the asexual intra-erythrocytic stages of the *Plasmodium* parasite. This highly metabolic developmental stage is responsible for all the clinical symptoms attributable to malaria [5]. Inside the erythrocyte malaria parasites use proteases to degrade 65–75% of host cell haemoglobin in a process that ultimately results in the release of free amino acids [6]. The initial steps in this process take place in a specialized digestive vacuole (DV) at pH 5.2 and involve aspartic proteases (plasmepsins I, II, IV and histo-aspartic protease), cysteine proteinases (falcipains 2, 2′ and 3), a metallo-protease (falcilysin) and a dipeptidyl aminopeptidase I, or cathepsin C [6], [7], [8]. The combined action of these proteases degrades the haemoglobin into small peptides or dipeptides that are suggested to be transported to the parasite cytosol for final processing by neutral exo-aminopeptidases [9], [10], [11]. Exo-aminopeptidases are essential in releasing amino acids from hemoglobin-derived peptides and are central to the growth and development of malaria parasites in the erythrocyte as the free amino acids are used by the parasite in protein synthesis [6], [12]. One of the pivotal aminopeptidases involved in this process is the *Plasmodium falciparum* alanyl aminopeptidase, termed *Pf*A-M1 because it belongs to clan MA, family M1 aminopeptidases. Inhibitors of this enzyme prevent both the growth of malaria parasites in erythrocytes in culture and *in vivo* in experimental mouse models and, hence, the enzyme is considered a prime target for the development of new anti-malaria drugs [11], [13].

The *Pf*A-M1 is encoded by single-copy gene, is a protein of 1,085 aa and molecular weight 126 kDa, but is detected as a 122-kDa protein in membrane fractions of malaria parasites [14]. *Pf*A-M1 orthologues of the various rodent malaria species (*P. berghei*, *P. chabaudi chabaudi*, and *P. yoelii*) share 70% identity and are most divergent at a large non-conserved N-terminal extension (194 aa), which contains 3 asparagine-rich low-complexity regions (LCRs) and a putative transmembrane domain. Most importantly, the catalytic machinery of all malaria M1 alanyl aminopeptidases is completely conserved [14]. Outside malaria, *Pf*A-M1 shows closest identity (35%) to the bacterial M1 aminopeptidases, which do not contain the extended N-terminal domain. However, the core structure of the clan MA, family M1 enzymes is well conserved in bacteria, plants, insects and mammals.

**Figure 1:**
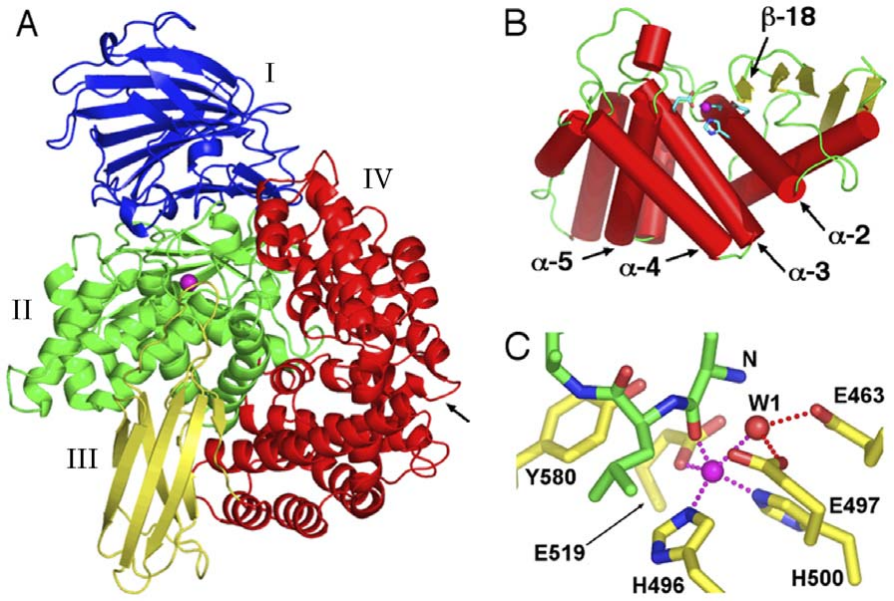
Structure of *Pf*A-M1 aminopeptidase N. A. Ribbon diagram of *Pf*A-M1 (PDB code 3EBH) coloured by domain: I (blue), II (green), III (yellow) and IV (red). Zinc ion depicted as purple sphere. Arrow indicates position of C-terminal channel opening. B. Catalytic domain II, with α-helices coloured red, loops green and β-strands yellow. Active site residues are shown in stick form with carbon cyan, oxygen red and nitrogen blue. Zinc ion depicted as purple sphere. The N-terminal lobe consisting of a 5-stranded β-sheet and α-helices 1–2 is to the right, the C-terminal lobe comprising α-helices 3–8 is to the left. The active site occurs in the cleft between the N- and C-terminal lobes. C. Ligand-bound active site. Active site of the starting structure of the ligand-bound complex used in the simulation. Ligand and active site residues are shown in stick form with carbon green (ligand) or yellow (*Pf*A-M1), oxygen red and nitrogen blue. The nucleophilic water is labelled “W1” and the ligand N-terminal amino nitrogen “N”. Hydrogen bonds and metallo bonds are shown as red and purple dotted lines, respectively.

A truncated form of the *Pf*A-M1 lacking the unique N-terminal extension (residues 195–1085, correlating with the start of the M1 aminopeptidase of *Escherichia coli* PepN) was expressed as a functional recombinant form in *E. coli* and the purified enzyme shown to exhibit physico-biochemical characteristics identical to those of a naturally occurring soluble form of the *Pf*A-M1 enzyme in malaria cells. The X-ray crystal structure of this construct was recently determined [15] and confirmed that *Pf*A-M1 adopts the bacterial aminopeptidase N-fold [16], [17], [18]. Thus, *Pf*A-M1 comprises 26 α-helices and 26 β-strands arranged in four domains, domain I–IV (Fig. 1A): an N-terminal domain (196–391, I), a catalytic domain (392–649, II), a middle domain (650–746, III), and a C-terminal domain (747–1084, IV). The N-terminal domain forms a β-sandwich structure composed of three ß-sheets. The catalytic domain, which forms an interface with all three other domains, is an α/β structure comprising a five-stranded β-sheet and eight α-helices. Like other M1 aminopeptidases, the enzyme contains a single catalytic zinc ion in the active site.

The active site is found in a groove that consists of one β-strand and three α-helices; β-strand 18 and α-helix 5 form each sidewall, and α-helices 2 and 3 serve as the base (Fig. 1B). The site contains the zinc-binding motif H^496^EYFHX_17_KE^519^ and the well-conserved G^490^AMEN motif involved in substrate recognition [16], [17], [18]. The catalytic zinc ion is coordinated by the Nε2 atoms of His-496 and His-500, the carboxyl O atom of Glu-519, and a water molecule that acts as the nucleophile that attacks the carbonyl carbon of the substrate (Fig. 1C). This water molecule forms a metallo-bond with the zinc ion and is also coordinated by Glu-497 and Glu-463.

There are two openings on the surface of the *Pf*A-M1 enzyme that connect to the active site cavity. The first opening (N-terminal channel) comprises a shallow 8 Å-long groove at the junction of domains I and IV. The second and larger opening (C-terminal channel) is a 30 Å-long channel that is formed by the C-terminal domain IV, which comprises 8 pairs of α-helices arranged in 2 layers to form a cone-shaped super-helical structure; this domain also interacts with the catalytic domain II. We have proposed that hemoglobin-derived peptides access the buried active site *via* the 30 Å long C-terminal channel and that hydrolytically released amino acids exit by the narrow N-terminal channel.

In order to investigate both the binding mode of a natural peptide ligand as well as the possible effect of ligand binding on the global dynamics and conformation of *Pf*A-M1, we performed two 75 ns unrestrained all-atom MD simulations of *Pf*A-M1; one of the enzyme without substrate (apo-enzyme state) and the other of the enzyme complexed with a four-residue peptide, Phe-Leu-Ala-Ser (ligand-bound state). This ligand sequence was chosen because (a) the coordinates of the first two residues can be derived from the X-ray structure of bestatin-bound *Pf*A-M1 (PDB code 3EBH), and (b) the last two C-terminal residues complete a tetrapeptide sequence found in human haemoglobin. Our studies provide new insights into the structure and catalytic mechanism of *Pf*A-M1, which are discussed in terms of the enzyme's pivotal function in the malaria parasite.

## Results and Discussion

### Interactions of the active site zinc cation reveal the basis of pentahedral coordination

The zinc divalent cation in the active site of *Pf*A-M1 poses a technical problem in MD simulations. This is because, whilst X-ray structures indicate tetrahedral or pentahedral coordination of the active site zinc [19], [20], use of the standard CHARMM forcefield parameters for zinc in MD simulations of *Pf*A-M1 resulted in hexacoordinated zinc (data not shown). This problem was solved by the cationic dummy atom model of Zn-ligand interactions [21]. In this model, four cationic dummy atoms, each with a charge of +0.5e, are tetrahedrally bonded to a central zinc atom, thus mimicking Zn's 4s4p^3^ vacant orbitals, and imposing the requisite orientational requirement for the ligands. The central zinc atom is assigned only vdW parameters, its +2e charge being distributed among the four dummy atoms, which interact with other atoms in the protein only via electrostatic interactions. Here we empirically modified the cationic dummy atom model for use with our MD code NAMD 2.6, the CHARMM27 forcefield and the *Pf*A-M1 protein (see ‘Methods’).

Pentahedral coordination of the zinc cation was maintained throughout the simulations of both the apo- and ligand-bound *Pf*A-M1 and was the same in both (Fig. 1C) with the only difference being a second water molecule occupying approximately the same position in apo-*Pf*A-M1 as a backbone carbonyl oxygen in the ligand-bound complex. Table 1 shows the average distances of the Zn-coordinating atoms to the central zinc atom in the simulations and for comparison, those from X-ray structures of *Pf*A-M1 and related N aminopeptidases from *E. coli* and *Neisseria meningitidis*. The mean distances between the Zn and its coordinating atoms in the simulations are in good agreement with the crystal structures. Indeed, the results match the crystal structures in this respect as well or better than do results from alternative schemes used for accounting for Zn coordination in MD simulations [21], [22], [23].

**Table 1:** Distances between the Zn atom and its coordinating atoms^a^.

	E519 oε1	H496 nε2	H500 nε2	Water 1 O	Water 2 O	Ligand O^b^
X-ray^c^	2.03±0.04	2.06±0.03	2.06±0.04	2.12±0.14	-	-
Apo^d^	2.04±0.04	2.09±0.03	2.10±0.03	2.05±0.04	2.29±0.15	-
Ligand^d^	2.05±0.04	2.10±0.03	2.08±0.03	2.17±0.14	-	2.05±0.09

a Mean values and standard deviations are in Å.

b Distance between Zn and the ligand backbone carbonyl oxygen of the scissile bond.

c Mean values and standard deviations obtained by averaging over 9 PDB structures of aminopeptidase N: *P. falciparum* (3EBH, 3EBI, 3EBG; [15]), *E. coli* (2ZXG; [24]), *E. coli* (2HPT, 2HPO; [17]), *E. coli* (2DQM, 2DQ6; [16]), *N. meningitidis* (2GTQ; [18]). The protein atom-Zn distances are the means of 9 measurements, while the water molecule distance is the mean distance measured from 4 unliganded structures.

d From PfA-M1 simulations, distances sampled every 50 ps over the 75 ns simulations.

Pentahedral coordination of the catalytic Zn was observed in crystal structures of a number of inhibitor-bound complexes of aminopeptidase N [16], [17], [18], [24]. Pentahedral coordination of the catalytic Zn is required to bind the transition state of the hydrolysis reaction, which forms upon nucleophilic attack by a water molecule at the carbonyl carbon of the substrate. Inhibitors such as bestatin mimic the transition state of the hydrolysis reaction by containing two oxygen atoms appropriately deployed to account for the backbone carbonyl oxygen of the ligand and the oxygen of the nucleophilic water. An interesting feature of the Zn coordination observed in all inhibitor-bound M1 aminopeptidase crystal structures reported is that, while the three protein atoms that coordinate the Zn are distributed in an approximately regular tetrahedral geometry, the two oxygen atoms of the inhibitor are located on opposite sides of the point corresponding to the fourth apex of a regular tetrahedron. This is shown in Table 2, where the angles formed by the Zn-coordinating atoms are tabulated for comparison between the crystal structures and the simulations.

**Table 2:** Angles between the Zn atom and its coordinating atoms^a^.

	E519 oε1H496 nε2	H496 nε2H500 nε2	H500 nε2E519 oε1	Water 1 O	O-Zn-O^b^
X-ray^c^	109.7±2.4	101.2±3.2	102.2±3.2	134.3±14.2	64.9±10.5
Apo^d^	109.8±8.7	97.7±4.1	102.0±6.6	-	82.2±6.9
Ligand^d^	110.0±5.7	99.7±4.8	96.6±4.5	-	76.4±3.2

a Mean values and standard deviations are in degrees.

b For the crystal structures, the angle between the Zn-coordinating oxygen atoms of the bound inhibitor was measured, for the apo simulation the angle between the Zn-coordinating oxygen atoms of the two proximal water molecules was measured, and for the ligand-bound simulation, the angle between the Zn-coordinating oxygen atoms of the nucleophilic water and the ligand carbonyl oxygen atom was measured.

c Mean values and standard deviations obtained by averaging over 9 PDB structures of aminopeptidase N: *P. falciparum* (3EBH, 3EBI, 3EBG; [15]), *E. coli* (2ZXG; [24]), *E. coli* (2HPT, 2HPO; [17]), *E. coli* (2DQM, 2DQ6; [16]), *N. meningitidis* (2GTQ, [18]). The protein atom-Zn angles are the means of 9 measurements, the water 1 angle is the mean distances measured from 4 unliganded structures. The O-Zn-O angle is the mean from 5 inhibitor-bound structures.

d From PfA-M1 simulations, angles sampled every 50 ps over the 75 ns simulations.

The irregularity of the penta-coordinate geometry of the Zn coordination observed in the inhibitor-bound crystal structures could perhaps be due to steric restrictions placed on the configuration of the inhibitor's Zn-coordinating oxygen atoms, although, the crystal structures of the unliganded protein do not support this idea. Thus, although the Zn is tetrahedrally coordinated in apo-enzyme structures, the single Zn-coordinating water molecule found in each structure is displaced to one side of the point corresponding to the apex of a regular tetrahedron, with respect to the other three coordinating atoms, similarly as observed for the ligand oxygen atoms in the inhibitor bound structures. This is shown in Table 2 by the average of the largest angle between the coordinating water and the coordinating protein atoms in four apo structures of M1 aminopeptidases. Notably, when two apo-enzyme structures of the *E. coli* aminopeptidase N from different studies [16], [17] are structurally overlaid, the Zn-coordinating water molecules from each structure also are found to be distributed to opposite sides of the point representing the apex of a regular tetrahedron, with respect to the three protein ligands (Fig. S1). Moreover, analogously to the Zn-coordinating oxygen atoms of the inhibitor in the bestatin-bound structures, one water molecule is hydrogen-bonded to Tyr-580, while the other forms hydrogen bonds to Glu-463 and Glu-497 (*Pf*A-M1 numbering).

Table 2 shows the mean angles between the Zn atoms and its coordination atoms from the simulations. The data indicate that the simulations reproduce the geometry of the pentahedral coordination of the Zn observed in the crystal structures very well. In the simulations, the irregular pentahedral coordination geometry occurs because, whilst three of the apex atoms of the Zn cation associate closely with one or other of the coordination atoms from the protein, the fourth apex atom is “shared” by the non-protein coordination atoms. This is illustrated in Table S1, which shows the mean distances of the Zn coordinating atoms to the proximal apex atom of the Zn cation.

As discussed above, in the crystal structures of *E. coli* apo aminopeptidase N, only one water molecule is resolved in the Zn coordination shell. Notably however, in the structure of apo-*Pf*A-M1, a second water molecule is located 2.62 Å from the Zn. This second water forms hydrogen bonds to Glu-463 and Glu-497 and approximately occupies the position of the nucleophilic water, as inferred from structures of inhibitor-bound aminopeptidase N (Fig. 1C). In the simulation of the apo-enzyme state, two water molecules were found to coordinate the Zn (Table 1). The water molecule occupying the site approximately equivalent to the ligand carbonyl oxygen (W2; Water 2 in Table 1) was found to exchange 16 times during the simulation, and to be situated significantly further away from the zinc atom than the water molecule occupying the position of the nucleophile (Table 1), which only exchanged once. The higher frequency of exchange of W2 is consistent with the fact that it must make way for the ligand carbonyl oxygen upon ligand binding. The observation from both the simulations and crystal structures that the non-protein oxygen atoms consistently migrate to one of two defined loci, indicates that the active site is structured both sterically and electrostatically to induce the irregular pentahedral coordination observed. Together, the data suggest that the penta-coordinate geometry of the Zn occurs because the two non-protein oxygen atoms share one of the Zn's vacant orbitals.

### Active site residue interactions: insights into the catalytic mechanism

**Figure 2:**
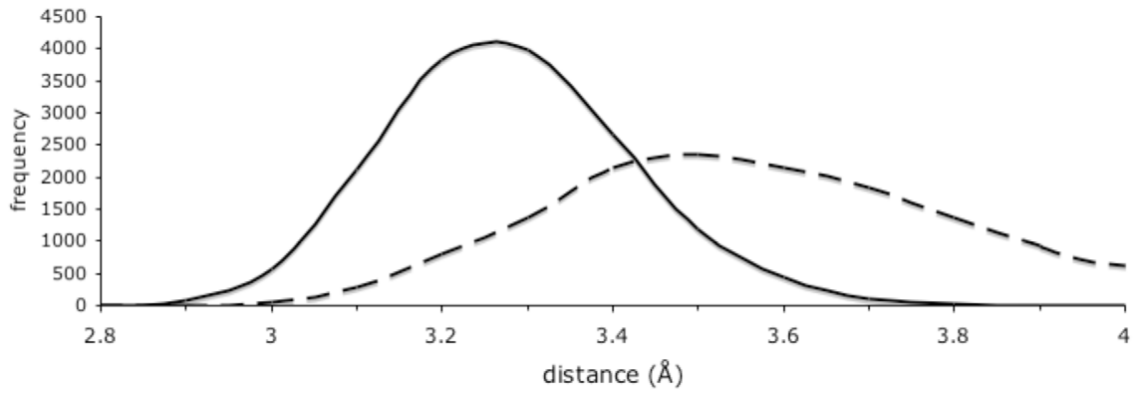
Approach profile of the nucleophilic water and Tyr-580 hydroxyl oxygens to the ligand carbonyl carbon. Smoothed histogram plots of distances to the ligand carbonyl carbon measured in 0.1 Å bins every 50 ps over the 75 ns simulation of the ligand-bound complex. Solid line, distances to nucleophilic water oxygen; dashed line, distances to Tyr-580 hydroxyl oxygen.

The simulation of the ligand-bound protein provided unique insights into the Michaelis complex, which differed in significant ways from the transition state approximated by the inhibitor-bound crystal structures. This revealed how the pentahedral coordination of the catalytic Zn brings the nucleophilic water into close proximity with the substrate carbonyl carbon; this is illustrated in Fig. 2, which shows a frequency histogram of the distances of the putative nucleophilic water oxygen to the scissile carbonyl carbon. The combined van der Waals radii of these two atoms in the CHARMM27 forcefield is 3.77 Å. In the simulation, the average distance between these atoms was 3.23 Å, whilst more than 18% of sampled distances were less than 3.1 Å, with a closest measured approach of 2.75 Å. Thus, the simulations suggest that pentahedral coordination of the catalytic Zn occurs in the ground (reactant) state of the hydrolysis reaction, and facilitates progress to the transition state by orienting the reactants i.e. directing the uncoordinated lone pair electrons of the nucleophilic water oxygen toward the substrate carbonyl carbon, and bringing them into close proximity.

An interesting feature of the active site during the ligand-bound simulation is the interactions of residue Tyr-580. From crystal structures of inhibitor-bound *E. coli* aminopeptidase N, it was inferred that the Tyr-580 hydroxyl moiety binds the substrate P1 carbonyl oxygen in the tetrahedral transition state of the hydrolysis reaction [17]. In our simulation of the ligand bound complex, it was observed that the mean distance between the Tyr-580 hydroxyl oxygen and the substrate P1 carbonyl carbon was 3.56 Å, while the mean distance between the Tyr-580 hydroxyl oxygen and the substrate P1 carbonyl oxygen was greater at 3.88 Å. The approach profile of the Tyr-580 hydroxyl oxygen and the substrate carbonyl carbon reveals a broad distribution of distances with closest approaches down to under 3 Å (Fig. 2). The close proximity of the Tyr-580 hydroxyl oxygen and the substrate carbonyl carbon in the Michaelis complex may facilitate progression to the transition state, aiding the polarisation of the substrate carbonyl O-C bond by inducing and stabilizing increased positive charge on the carbon, thus acting to destabilize the ground (reactant) state. This scenario supports the role proposed for the equivalent residues in both M1 aminopeptidases [17] and thermolysin [25] in decreasing the activation energy of the hydrolysis reaction, in contrast to alternative schemes in which it is suggested to protonate the P1′ amide [26], [27], [28].

**Figure 3:**
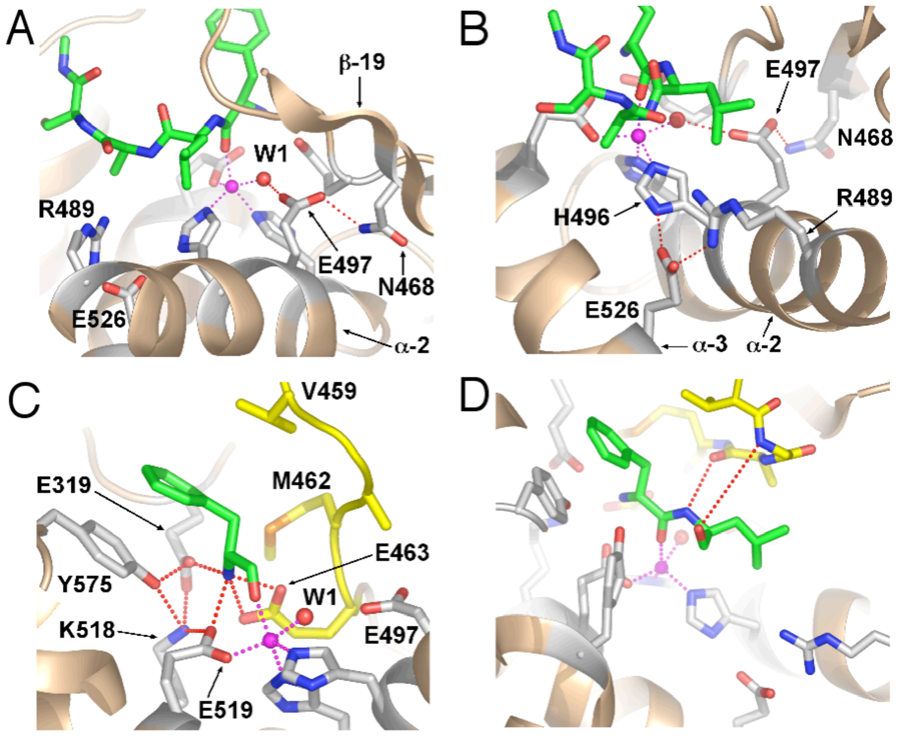
Changes in interactions of active site residues in the ligand-bound complex. Diagrams of the final frame (t = 75 ns) from the simulation of the ligand-bound complex. Ligand and active site residue sidechains are shown in stick form with carbon green (ligand) or light grey (*Pf*A-M1), oxygen red, nitrogen blue and sulphur orange. Zn ion depicted as a purple sphere. Metallo bonds shown as purple dotted lines and hydrogen bonds as red dotted lines. A. Interaction of Glu-497 with Asn-468. B. Interaction of Glu-526 with His-496 and Arg-489. C. Network of interactions with the ligand N-terminal amino nitrogen and Tyr-575. GAMEN motif residues coloured yellow. D. Interaction of GAMEN backbone amide of Gly-460 and main-chain carbonyl oxygen of Ala-461 with the ligand. The Gly-460 amide-P1′ carbonyl oxygen hydrogen bond, present in the crystal structure, is lost due to conformational changes in the loop immediately N-terminal to Gly-460. GAMEN residues shown in stick form with carbon atoms coloured yellow.

Other observations from the simulations shed important light on the details of the catalytic mechanism and the roles of active site residues. In inhibitor-bound crystal structures, Glu-497 is within hydrogen-bond distance of the ligand oxygen expected to mimic that of the nucleophilic water, and is postulated to act as the catalytic base of the hydrolysis reaction in aminopeptidase N [17], [29], as well as other enzymes from the same superfamily [28], [30], [31]. In our ligand-bound, but not the apo-enzyme simulation, the Glu-497 sidechain undergoes a conformational change from the disposition observed in crystal structures and the simulation starting structure (Fig. 1C) to form a hydrogen bond between one of its sidechain carboxylate oxygens and the sidechain of residue Asn-468 whilst still maintaining a hydrogen bond to the nucleophilic water (Fig. 3A). These observations indicate that the Michaelis complex differs from the transition state in this respect and are consistent with the idea that Asn-468 has an ancillary role in the catalytic mechanism, orienting and activating the Glu-497 carboxylate moiety. It is important to note that Asn-468 is highly conserved in the aminopeptidase N family, including those expressed by malaria parasites of humans (*P. falciparum* and *P. vivax*), monkeys (*P. knowlesi*) and mice (*P. berghei*, *P. chabaudi* and *P. yeoli*) (Fig. S2). Also consistent with this proposal is that in the orientation observed in the ligand-bound simulation, Glu-497 is in a more buried, hydrophobic environment (Fig. 3A,B) than in the starting structure where it is in closer proximity to polar and ionized moieties (Fig. 1C). This hydrophobic environment would facilitate the protonation of the Glu-497 carboxyl sidechain by the nucleophilic water, since the ionized state of the carboxylate would be energetically less favourable here.

Another novel insight from the simulations with important implications for the catalytic mechanism is the observation that Glu-463 is also involved in binding the nucleophilic water; in the ligand-bound simulation the average distance of the hydrogen bond to the nucleophilic water was 2.57±0.08 for Glu-497 and 2.66±0.12 Å for Glu-463. Glu-463 is part of the highly conserved GAMEN motif (see below), and was previously implicated in both ligand binding and catalysis by biochemical data [29], [31]. Although crystal structures elucidated its role in ligand binding by showing its interaction with the ligand N-terminal amino nitrogen [14], [17], [31] the structural basis for its role in catalysis remained unclear. Our simulation is consistent with the biochemical data in demonstrating how Glu-463 plays a role in catalysis i.e. by binding the nucleophilic water and plausibly the transition state (see below), and also further supports its role in ligand binding through its ionic interaction with the ligand N-terminal nitrogen (Fig. 3C).

**Figure 4:**
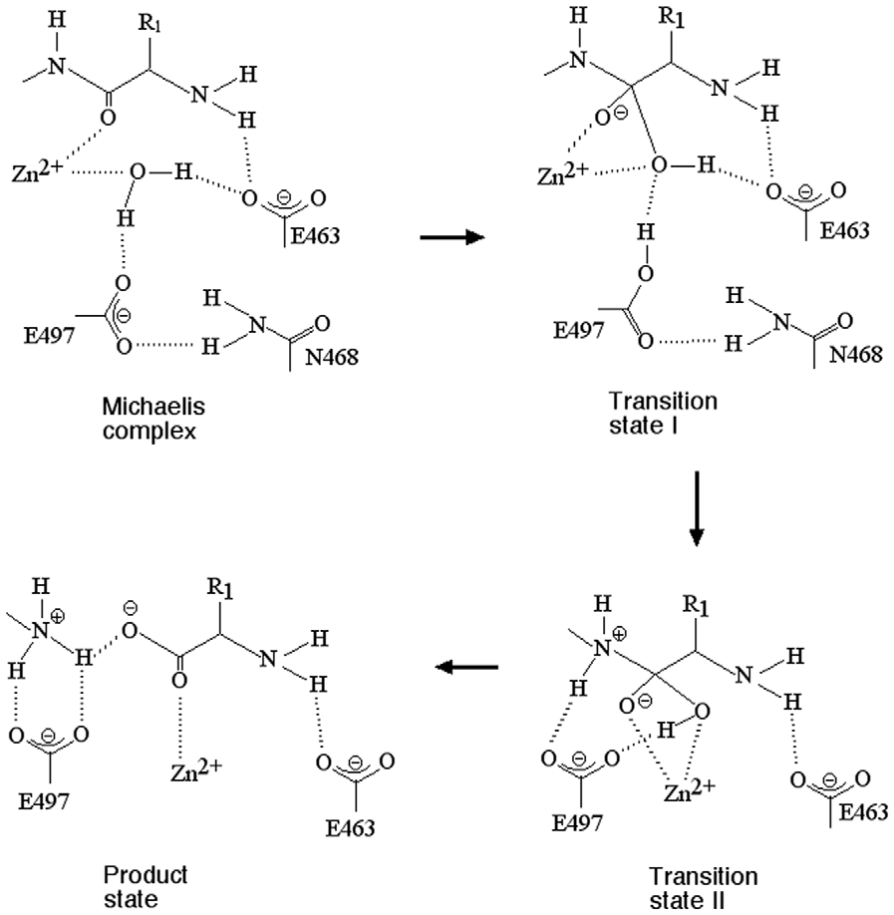
Scheme for the catalytic mechanism of M1 aminopeptidases.

A scheme for the catalytic mechanism is proposed in Fig. 4. This scheme is essentially the same as that proposed previously [17], but includes further articulation of the sequence of events inferred from our data in this study. The hydrolysis of the peptide bond proceeds via a mechanism that comprises two sequential transition states, I and II, that are differentiated by the distinct conformations of Glu-497, which in turn correspond to the abstraction of the nucleophile proton and its subsequent transfer to the P1′ amide of the scissile bond. In transition state I, Glu-497 (activated by Asn-468) accepts the nucleophile proton and the tetrahedral intermediate forms, with Glu-463 aiding in its stabilization through the interaction with the hydroxyl moiety. In transition state II, formation of the tetrahedral intermediate has induced a conformational change that results in Glu-497 flipping around to transfer its proton to the P1′ amide. This conformation is envisaged to resemble that in the starting structure (Fig. 1C), where the Glu-497 carboxyl moiety is also able to bind the tetrahedral intermediate hydroxyl, and thereby play a role in facilitating the transfer of the hydroxyl proton to the P1′ amide upon collapse of the transition state.

### The GAMEN motif

In *Pf*A-M1, residues 460–464 form the so-called GAMEN substrate specificity motif common to many exopeptidases [14]. In M1 aminopeptidases, the residue positioned immediately preceding the GAMEN motif (Val459 in *Pf*A-M1) interacts with the sidechain of substrate residue P1 and likely influences substrate specificity. The GAMEN recognition motif residues also contribute hydrogen bonds to ligand binding main-chain amide of Gly-460, main-chain carbonyl oxygen of Ala-461 and with the side chain of Glu-463. We found that movement of N-terminal regions of the GAMEN motif, together with movement of the C-terminal residues of the substrate, resulted in breaking of the interaction between the Gly-460 amide and the backbone carbonyl oxygen of substrate residue P2′. The interaction between the main-chain carbonyl oxygen of GAMEN residue Ala-461 with the substrate P1′ backbone amide, remained intact throughout the simulation (Fig. 3D).

### The S1 pocket loop

During our simulation of the ligand-bound state we observed that marked alterations occurred in the region known as the S1 pocket loop, which accepts the P1 substrate residue (residues 570–575), resulting in altered interactions within the active site. Thus, rotation of the sidechain of Tyr-575, which together with Val-459 comprises the predominant interaction of the protease with the substrate P1 sidechain, resulted in the formation of hydrogen bonds between its hydroxyl group and Lys-518 and Glu-319. This broke the hydrogen bond between Glu-463 and Lys-518 observed in all crystal structures of aminopeptidase N, and resulted in the formation of bidentate hydrogen bonds between Glu-463 and substrate N-terminal amino group (Fig. 3C).

Notably, recent structural analyses of *Pf*A-M1 found significant conformational changes in residues 570–575 in response to binding derivatives of the inhibitor bestatin with larger P1 groups, Tyr(OMe) or Tyr(OBzl). In the case of Tyr(OBzl), the Cα atom of Glu572 moved by ∼5 Å, relative to that observed in the bestatin-bound structure, in order to accommodate the bulkier P1 sidechain. This study also found that the S1 pocket loop (residues 570–575) is mobile with high B-factors, in contrast to previous structural characterizations of *Pf*A-M1 homologues, which did not identify movement in the S1 pocket nor indicate any enhanced mobility of this region. Our simulations are consistent with the experimentally observed plasticity and mobility in the S1 pocket loop, with the Cα atom of Glu572 moved by 3 Å in the ligand-bound structure at t = 75 ns. In addition, our results suggest that differences may occur in this region between the Michaelis complex and the transition state, resulting in altered interactions between the protein and the ligand N-terminal amino group, not previously observed in crystal structures.

### Changes in the active site are coupled to global conformational and dynamic changes

**Figure 5:**
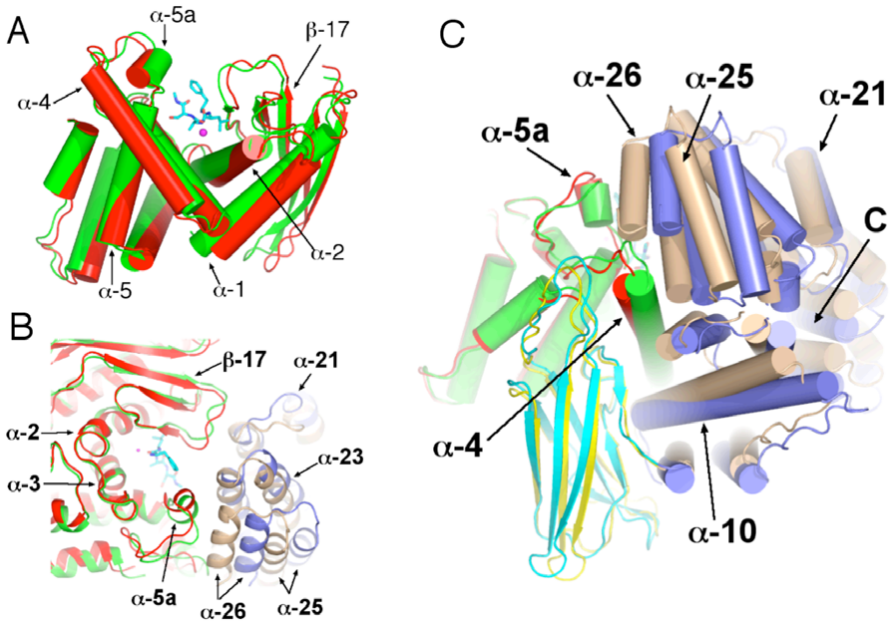
Conformational changes in the ligand-bound complex. Structural alignments of frames at t = 0 and t = 75 ns from the simulation of the ligand-bound complex. Frames aligned using Cα coordinates of domain II. Ligand shown in stick form with carbon cyan, oxygen red and nitrogen blue. Zn ion depicted as a purple sphere. Secondary structural elements as indicated. A. Catalytic domain II (t = 0 green, t = 75 red). C-terminal of α-helix 1 is indicated by the arrow. B. Small N-terminal channel opening. Catalytic domain II (t = 0 green, t = 75 red). Domain IV (t = 0 tan, t = 75 mauve). Domain I, which would partially occlude the view of the opening to the active site, has been removed for clarity. C. Coupling of conformational changes in catalytic domain II (t = 0 green, t = 75 red) with changes in domains III (t = 0 yellow, t = 75 cyan) and IV (t = 0 tan, t = 75 mauve). Opening to C-terminal channel indicated by “C”.

The altered interactions observed for residues within the active site that occurred during the simulation of the ligand-bound complex are associated with a global network of changes in the protein conformation. Internally, domains I and III maintained their starting conformations, with rms deviations from the crystal structure at 75 ns of 1.08 Å and 0.96 Å, respectively, for Cα atoms within each domain aligned separately. Domains II (catalytic domain) and IV (C-terminal domain) altered internally more so, with rms deviations of 1.37 Å and 1.65 Å, respectively. Within the catalytic domain II, the β-sheet within its N-terminal lobe, together with regions of the C-terminal α-helical bundle, formed a relatively rigid structure about which changes occurred. Most striking, α-helix 2, which contains residues HEYFH of the conserved zinc binding motif, pivots about its N- and C-termini, moving the HEYFH residues away from α-helix 3, which forms the other side of the base of the active site groove (Fig. 5A). This change was aided by concerted alterations in α-helix 1 and the loop at its C-terminus, and these changes allowed the interaction of Glu-497 with Asn-468, which is on β-strand 19 (see above and Fig. 4 regarding importance of this interaction in the catalytic mechanism).

**Figure 6:**
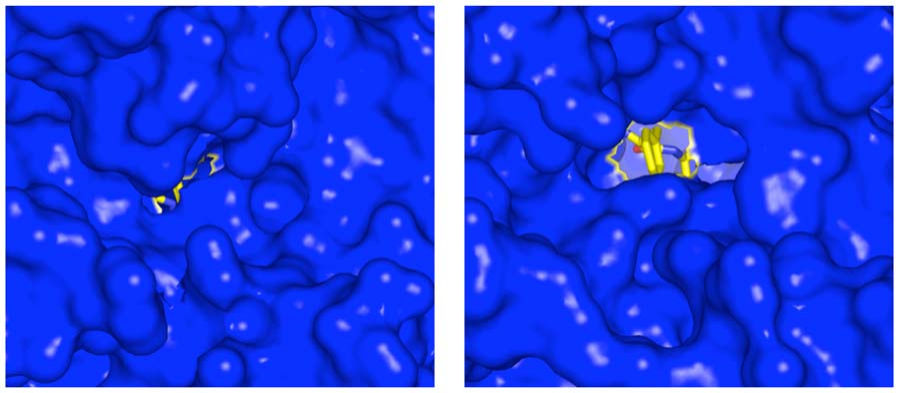
Opening of the N-terminal small channel. Surface representation (blue) of the starting structure (left) and the ligand-bound structure at t = 75 ns (right). Ligand is shown in stick form with carbon yellow, oxygen red and nitrogen blue. The N-terminal phenylalanine residue of the ligand is visible in the opened channel at t = 75 ns (right).

The other relevant change in catalytic domain II was with respect to α-helix 4, the C-terminus of which moves away from the active site. This occurs in concert with alterations in the loop joining its C-terminus to α-helix 5; this includes the S1 pocket loop, which encompasses α-helix 5a and the short loop joining it to α-helix 5 (Fig. 5A). Together with these changes, large changes in the two C-terminal α-helices of domain IV (25 and 26; Fig. 5B) occurred which resulted in significant enlargement of the N-terminal opening of the 8 Å-long channel to the active at the junction of domains I and IV by which freed amino acids leave (Fig. 6). In the apo-enzyme simulation, enlargement of the N-terminal small opening also occurred but to a lesser extent, and while the contribution from changes in domain IV α-helices 25 and 26 was greater, changes in the S1 pocket loop were less than in the liganded simulation.

### A flexible arm regulates peptide entry into the active site

**Figure 7:**
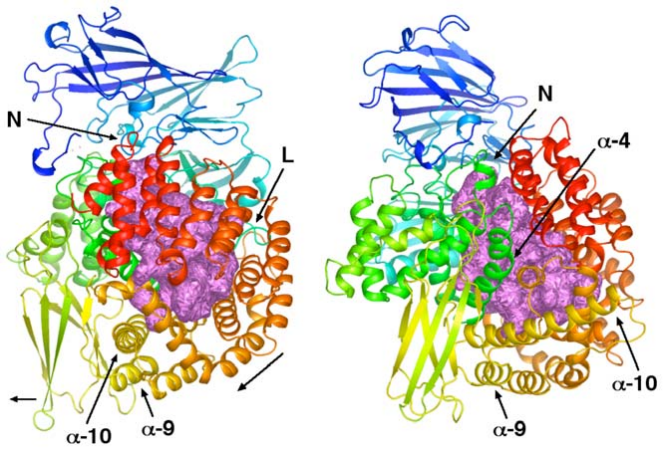
Propagation of conformational changes from the active site to domain III and α-helix 10. Two roughly orthogonal views of *Pf*A-M1 with secondary structural elements coloured in a spectrum pattern, blue to red, according to residue number. The internal chamber within the protein is shown in space filling representation and coloured violet. N-terminal channel indicated by an “N”. Left panel: Loop between β-strand 19 and α-helix 2 in domain II indicated by “L”. α-helices 11–19 are coloured orange through yellow and appear in the lower right quadrant. This region rotates in the direction of the oblique arrow. Domain III (lower left) moves in the direction of the small horizontal arrow. Right panel: α-helix 4 from domain II moves down and to the right, impinging on α-helix 10 at its N-terminus and also via α-helix 13.

Changes that occurred in the vicinity of the active site also occurred in concert with rigid-body motions of domain III and changes in α-helix 10 (residues 761–789) within domain IV. The C-terminal residues of α-helix 10 and the immediate downstream loop embody a flexible arm-like protuberance from the main body of the protein proximal to the opening of the 30 Å-long C-terminal channel. Over the course of the whole simulation, residues at the C-terminus of α-helix 10 (residues 785–795) undergo the greatest rms deviation of Cα atoms between the starting structure and final structure (Fig. S3A), and also undergo the greatest rms fluctuations of all residues with respect to the starting structure (Fig. S3B). This is true for the simulations of the ligand-bound, but not the apo-enzyme simulation (Fig. S3). Changes in α-helix 10 and domain III appeared to be transmitted from the catalytic domain around the outside of the large central chamber *via* two distinct routes. On one side, direct interactions occurred between α-helix 10 and domain III and α-helix 4 and the loop at its N- and C-termini (these interactions are illustrated in Fig. 5C). On the other side, changes that occurred at the N-terminus of α-helix 2 and the N-terminal loop joining it to β-strand 19 were coupled to rotational movement of α-helices in domain IV that ultimately influenced the positions α-helix 10 and domain III. These changes are illustrated in Fig. 7.

**Figure 8:**
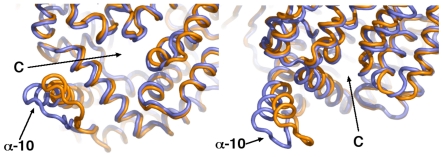
Concerted motions of the α-helix 10 C-terminal arm toward the large C-terminal channel. Two views of the overlayed maximum (orange) and minimum (slate) projection structures of PCA eigenvector 2 from the simulation of the ligand-bound complex. Opening to the C-terminal channel indicated by a “C”.

Elastic network (EN) analysis indicated that motions of residues at the C-terminus of α-helix 10 constitute the fundamental dynamic mode of the protein (Fig. S4). EN analyses the energetics of the protein's structure and detects global dynamic modes imprinted in the architecture; these often play functional roles [32]. Supporting the EN analysis, principal component analysis (PCA; see Methods) of the simulation trajectory revealed that during the simulation of the ligand-bound complex, cyclical concerted motions of the region of the C-terminus of α-helix 10 occur. In particular, the motion described by PCA eigenvector 2 included a large sweeping motion of the α-helix 10 ‘arm’ toward and away from the C-terminal large opening (Fig. 8). Motions in a direction transverse to this were also observed, indicating that the dynamics of this region are complex.

### Summary of observation and relevance to PfA-M1 function in malaria

Two 75 ns unrestrained all-atom MD simulations of *Pf*A-M1 were performed; one of the apo-enzyme state and the other of the protease complexed with a four-residue peptide. In order to reproduce the coordination of the active site zinc cation observed in crystal structures, the forcefield partial charges within the protein and ligand Zn-coordinating moieties were adjusted such as to preclude their substitution by a water molecule. The empirical adjustment of the forcefield partial charges to account for charge redistribution effects at the metal binding site is in line with recent studies addressing this problem in MD simulations [22], [23]. The modified forcefield very accurately reproduced the distances and geometry of the protein atoms chelating the catalytic Zn observed in crystal structures, and induced an irregular pentahedral coordination also consistent in terms of its geometry with crystal structures. Together with the structural data, the results indicate that the active site is structured sterically and electrostatically to induce an irregular pentahedral coordination, in which two non-protein oxygen atoms share one of the Zn's vacant orbitals. In the ligand-bound state, this geometry facilitates progress to the transition state by orienting the reactants and bringing them into close proximity.

The simulation of the ligand-bound state revealed that the conformation of the Michaelis complex differs in significant ways from that of the transition state, providing insights into the catalytic mechanism. Tyr-580, which binds the ligand carbonyl oxygen in the transition state, interacted more directly with the ligand carbonyl carbon in the Michaelis complex, suggesting it has a role in destabilizing the ground (reactant) state of the hydrolysis reaction. Residue Glu-463 maintains a hydrogen bond to the Zn-coordinating water located at the position of the attacking nucleophile, consistent with biochemical data indicating that this residue has a role in catalysis. Finally, we observed in the ligand-bound simulations that the conformation of the catalytic domain in the Michaelis complex differed from the apo-enzyme and the reported inhibitor-bound crystal structures, resulting in the formation of a hydrogen bond between Asn-468 and Glu-497, such that Glu-497 also maintained a hydrogen bond to the putative nucleophilic water. This configuration is consistent with the expected role of Glu-497 as the catalytic base, and locates the residue in a buried hydrophobic environment, which is energetically favorable to it accepting a proton from the attacking nucleophilic water. The results suggest a two-step catalytic mechanism, whereby formation of the tetrahedral transition state induces a conformational change in the catalytic domain such that Glu-497 rotates, enabling it to donate its proton to the leaving amide of the ligand, to complete the hydrolysis reaction (see Fig. 4).

The simulation of both the ligand-bound complex and apo-enzyme state revealed that changes in conformation in the catalytic domain are coupled to global conformational changes in the protein. These appear to be driven by changes in conformation at the N-terminus of α-helix 2 and at the C-terminus of α-helix 3, α-helix 4 and the downstream loop joining to α-helix 5. Near the active site, this resulted in opening of the shallow 8 Å-long N-terminal small channel located at the junction of domains I and IV which is expected to be the point of exit for the free amino acid following proteolysis. Conformational changes are also propagated from the catalytic domain toward α-helix 10 *via* two routes around the central cavity. The C-terminus of α-helix 10 and downstream loop proximal to the opening of the 30 Å-long C-terminal channel undergoes the largest fluctuations within the protein and EN analysis suggests that these are of major functional significance.

The large size of the internal 30 Å-long C-terminal channel invites questions as to its function; it could be capable of enclosing a continuous peptide that reaches the entrance at the C-terminal opening and beyond. It seems reasonable to suggest that the *Pf*A-M1 can digest peptides of 8–10 residues or more and that the function of the α-helix 10 arm is to move the peptide into the cavity as free amino acids are released at the N-terminal small opening. Indeed the PCA analysis of the simulation trajectories indicated that changes in the catalytic domain are correlated with changes in α-helix 10. Thus, after the N-terminal small channel opens to release product, the α-helix 10 could facilitate movement of the peptide ligand one amino acid into the cavity by acting in a ‘ratchet-like’ fashion. Alternatively, or additionally, the internal chamber may act as a kind of vestibule or ‘waiting room’, for smaller peptides that get shunted along toward the active site like an assembly line. Digestion of host hemoglobin within the digestive vacuole by a series of endopeptidases and exopeptidases likely yields peptides of various sizes but the exact size of peptides that are transported to the cytoplasm for further processing by the M1 aminopeptidase is not known. However, when human hemoglobin was incubated with lysates of *P. falciparum* digestive vacuole a series of discrete peptide fragments were generated with cleavage sites an average of 8.4 amino acids apart [10]. Nevertheless, we suggest there is a concerted conformational dynamic between the ‘ratcheted’ movements in α-helix 10, movements of peptides along the 30 Å-long C-terminal channel towards the active site, hydrolysis of the N-terminal substrate amino acid and its exit via the 8 Å-long N-terminal small channel and widening of the opening.

In showing how the Michaelis complex differs from the transition state conformation, the results provide insights that could aid in the design of agents to inhibit this class of enzyme that target the active site. In the case of *Pf*A-M1, however, due to its homology with various mammalian aminopeptidase N, drugs may need to be targeted to more sequence-diverse regions to reduce off-target side effects. Our results indicate that the large central cavity, particularly areas close to the active site that bind the P1-3′ substrate sidechains could be fruitful areas of investigation in this regard. In addition, the results support the flexibility and plasticity of the S1 pocket loop inferred from a recent structural analysis [33], and thus support the idea that this pocket may also be exploited in the development of new therapeutic agents against malaria.

## Methods

Two 75 ns all atom MD simulations of *Pf*A-M1 were performed, one of the unliganded (apo-enzyme) state and the other in which the protein was complexed with a four-residue peptide bound in the active site. Starting coordinates were the 1.65 Å resolution crystallographic structure of *Pf*A-M1, complexed with the inhibitor bestatin [14]; PDB code 3EBH). Seven engineered mutations were reversed to the wild type sequence, as indicated in the PDB file header (3EBH). Initial coordinates for a template peptide substrate (PHE-LEU-ALA) were derived from coordinates of the bestatin inhibitor bound in the active site. This initial peptide was minimized while bound to *Pf*A-M1 using the equilibration protocol described below. The equilibrated peptide was then extended by one serine residue at its C-terminus, using the coordinate generation function in the psfgen program [34], and then re-equilibrated. Finally, the C-terminal residue of the substrate was N-methylamidated and re-equilibrated; the resultant four-residue peptide PHE-LEU-ALA-SER was used in the simulation of the ligand-bound complex. The protein was optimally oriented to minimize cell volume [35] and solvated in a truncated octahedral periodic cell with a minimum of 20 Å between periodic images of the protein; the system was neutralized with a 0.2 M NaCl solution.

MD simulations were carried out with NAMD 2.6 [34] using the CHARMM27 force field with φ/ψ cross-term map corrections [36]. Water molecules were simulated with the TIP3P model [37]. Simulation conditions were maintained at 1.0 atm constant pressure by the Nosé-Hoover Langevin piston method [38], [39] and at 310 K constant temperature by Langevin dynamics with a damping coefficient at 5 ps^−1^. The time step used for the simulations was 1 fs. A cutoff of 11 Å, with a switching function between 9.5 and 11 Å, was used for short-range non-bonded interactions. Long-range electrostatic interactions were computed using the particle mesh Ewald method [40] with a grid density of approximately 1/Å. The mollified impulse method [41] was used allowing a multiple time-stepping algorithm, with interactions involving covalent bonds computed every time step, short-range non-bonded interactions every two time steps, and long-range electrostatic forces every 6 time steps.

The catalytic zinc was simulated using a five-atom tetrahedral zinc divalent cation, comprising a centre atom and four identical atoms located at the apices of a regular tetrahedron. Parameters for the bonded interactions of the zinc apex atoms were as for the dummy atoms in [21]. A mass of 3 and a charge of +0.5*e* was assigned to the apex atoms. Dummy atoms are not supported in NAMD 2.6 and a Leonard-Jones parameter of ε = 0.001 kcal/mol, r = 0.1 Å was assigned to the apex atoms. A 1fs timestep was required for calculation of bonded interactions of the apex atoms. For the central zinc atom, the standard CHARMM27 parameters were used, but with a charge of zero.

In preliminary simulations using this model, water molecules eventually replaced the two histidine Nε2 atoms and the ligand P1 carbonyl oxygen in the first coordination sphere of the zinc ion. Increasing the partial charge on the two Zn-coordinating histidine Nε2 atoms from −0.7 to −0.92, and on the ligand P1 carbonyl oxygen from −0.51 to −0.76, was minimally required to maintain these atoms within the Zn first coordination sphere. For the Zn-coordinating histidines, the residual charge was distributed over the histidyl moiety, while for the ligand the charge of the carbonyl carbon of the scissile C-O bond was increased from +0.51 to +0.76 to maintain electrical neutrality. Adjustment of the forcefield partial charges to account for polarisation effects at the metal binding site is in line with recent studies addressing this problem in MD simulations [22], [23]. In addition, increased polarisation of the ligand scissile C-O bond is consistent with the expected catalytic mechanism. To marginally increase the average distance between the coordinating carboxylate oxygen of E519 and the Zn atom, bringing it closer to that observed in crystal structures of related enzymes, Leonard-Jones interactions between all zinc atoms and glutamate and aspartate sidechain oxygens were adjusted using the NBFIX facility, such that type OC atoms (carboxylate oxygens) were parameterised at ε = 0.1521 kcal/mol, r = 3.54 Å, which are the nonbonded parameters for hydroxyl oxygens in the CHARMM27 forcefield.

During the equilibration phase of the ligand-bound complex, harmonic restraints with a force constant of 10 kcal/mol were applied between the proximal zinc apex atom and the sidechain carboxylate oxygen of Zn-coordinating residue 519, between the ligand scissile bond Zn-coordinating carbonyl oxygen and the central zinc atom, and between the active site Zn-coordinating nuclephilic water oxygen and the central zinc atom. The solvated starting structure was minimized using conjugate gradient minimization to a 0.5 kcal/(mol·Å) r.m.s. gradient with all enzyme heavy atoms fixed. The unrestrained atoms were then further minimized during a 50 ps molecular dynamics run at 310 K. This starting model was then minimized with harmonic positional constraints on the NCαCO backbone of the protein and Cα atoms of ligand residues 3–4. A 100 kcal/(mol·Å^2^) force constant was used to minimise the system to a 0.5 kcal/(mol·Å) r.m.s. gradient. The harmonic positional constraints were gradually removed by subsequent minimizations to a 0.1 kcal/(mol·Å) r.m.s. gradient, scaling the initial force constants by factors of 0.5, 0.15, 0.05, and 0. The minimized structure was then heated from 50 K to 310 K in steps of 25 K using velocity reassignment during a 30 ps molecular dynamics run. For the ligand-bound system, the force constant of the harmonic distance restraints imposed on the Zn atoms during the above equilibration were reduced to 5 kcal/mol, and a short 0.5 ns MD run was performed. The two equilibrated systems were then used for production runs of 75 M integration steps with no restraints. Both simulations remained stable to completion. For analysis, coordinates were recorded every 5000 timesteps (5 ps).

Principal component analysis (PCA) of the Cα atom coordinate trajectory was used to identify and characterise global conformational transitions. PCA defines a set of eigenvectors (EVs) derived from the matrix of pairwise correlated motion of atoms. EVs are ranked according to the amplitude of the protein motions they describe and in general, the first 1 to 4 EVs account for the most concerted 50% or more of protein fluctuations. PCA of the simulation α-carbon atom trajectories was performed using the GROMACS package [42].

Elastic network analysis was performed on the *Pf*A-M1 structure (3EBH) using the online facility [43] available at: http://ignm.ccbb.pitt.edu/, using the Anisotropic Network Model (ANM; [44]) with default settings. All structural figures were prepared using PyMol (http://www.pymol.org/). The programs VMD [45], (available at http://www.ks.uiuc.edu/Research/vmd/), Xplor-NIH [46] and Simulaid (http://atlas.physbio.mssm.edu/~mezei/) were used in the preparation and analysis of the simulation system.

## Supporting Information

Figure S1 **Loci of Zn-coordinating water molecules in crystal structures of aminopeptidase N.** Structural alignment of the *E. coli* pepN active site from two apo structures: 2HPO (green; [16]) and 2DQ6 (cyan; [17]). Zn coloured blue (2HPO) and red (2DQ6). Residues numbered as in *Pf*A-M1. Metallo bonds in 2HPO shown by blue dotted lines. Hydrogen bonds to water molecules indicated by red dotted lines.
(TIF)

Figure S2 **Primary sequence alignment of M1 aminopeptidases from various** ***Plasmodium*** **species.** Identical residues are highlighted in red and conservatively substituted amino acids are shown in red text. The putative transmembrane domain is boxed in orange and the GAMEN substrate-recognition motif is boxed in black. The zinc-binding motif is underlined and the catalytic residues are indicated with arrowheads. Asn-468 and Tyr-580 are indicated (asterisks).
(TIF)

Figure S3 **Per residue changes during the simulations.** A. Per residue rms deviation relative to the starting structure after alignment using coordinates of the Cα atoms of domain II (392–649). Ligand-bound complex blue, apo red. B. Per residue rms fluctuations relative to the starting structure after alignment using coordinates of all Cα atoms. Ligand-bound complex blue, apo red.
(TIF)

Figure S4 **Elastic network analysis of** ***Pf*** **A-M1.** Per residue fluctuations due to slowmodes 1–3 from the ANM analysis of *Pf*A-M1 (PDB 3EBH). Units of fluctuations are relative only. Mode 1, blue; mode 2, red; mode 3, yellow.
(TIF)

Table S1 **This table shows the mean distances between the Zn apex atoms and the proximal coordinating atoms.**
(DOCX)
