# Supplementary figures and images for: The Plasmodium falciparum Malaria M1 Alanyl Aminopeptidase (PfA-M1): Insights of Catalytic Mechanism and Function from MD Simulations
Source: PLoS One. 2011 Dec 21;6(12):e28589. doi: 10.1371/journal.pone.0028589 (PMC3244404; doi:10.1371/journal.pone.0028589)

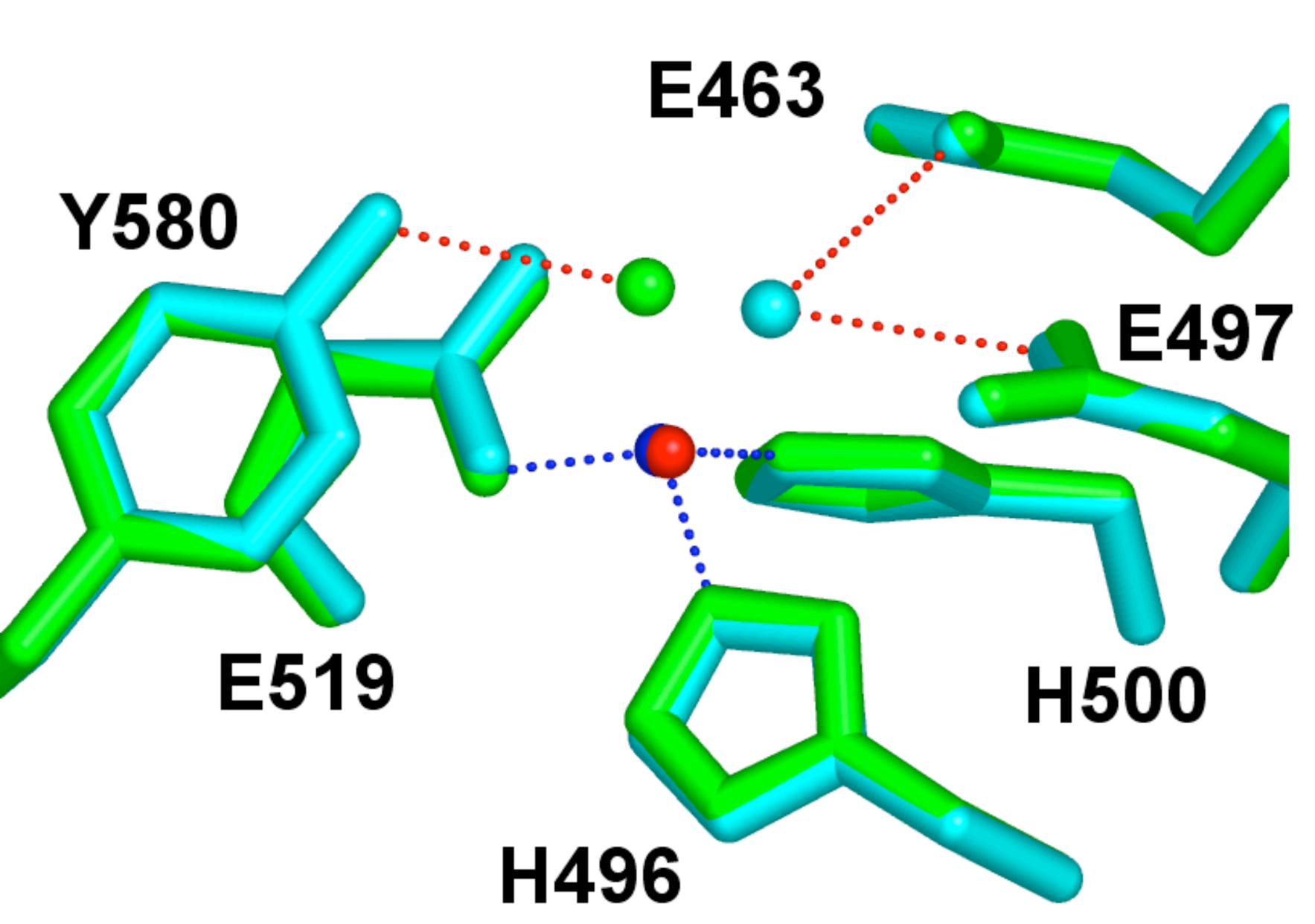

Supplement: Figure S1 — Loci of Zn-coordinating water molecules in crystal structures of aminopeptidase N. Structural alignment of the E. coli pepN active site from two apo structures: 2HPO (green; [16]) and 2DQ6 (cyan; [17]). Zn coloured blue (2HPO) and red (2DQ6). Residues numbered as in PfA-M1. Metallo bonds in 2HPO shown by blue dotted lines. Hydrogen bonds to water molecules indicated by red dotted lines. (TIF) [file pone.0028589.s001.tif]

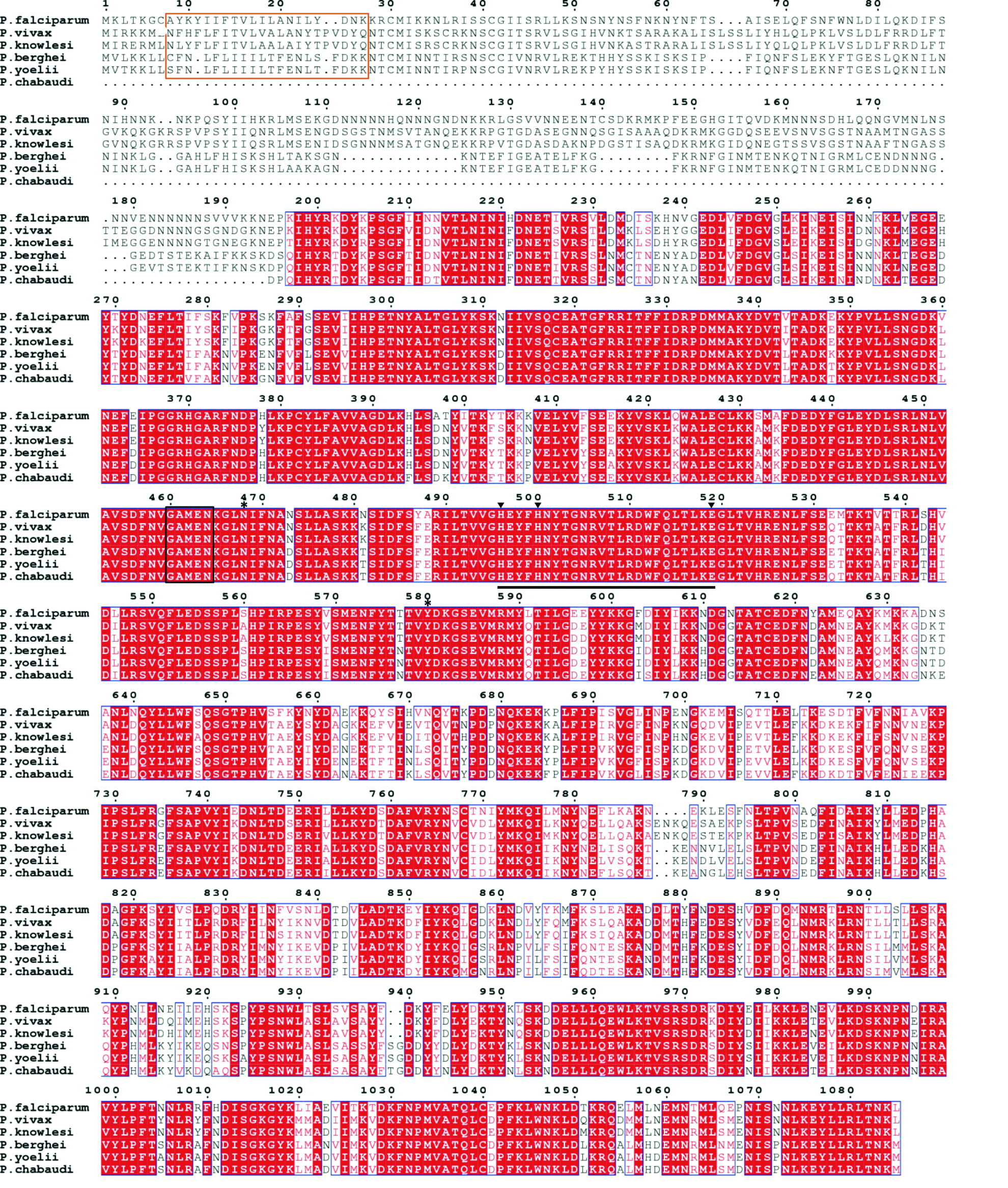

Supplement: Figure S2 — Primary sequence alignment of M1 aminopeptidases from various Plasmodium species. Identical residues are highlighted in red and conservatively substituted amino acids are shown in red text. The putative transmembrane domain is boxed in orange and the GAMEN substrate-recognition motif is boxed in black. The zinc-binding motif is underlined and the catalytic residues are indicated with arrowheads. Asn-468 and Tyr-580 are indicated (asterisks). (TIF) [file pone.0028589.s002.tif]

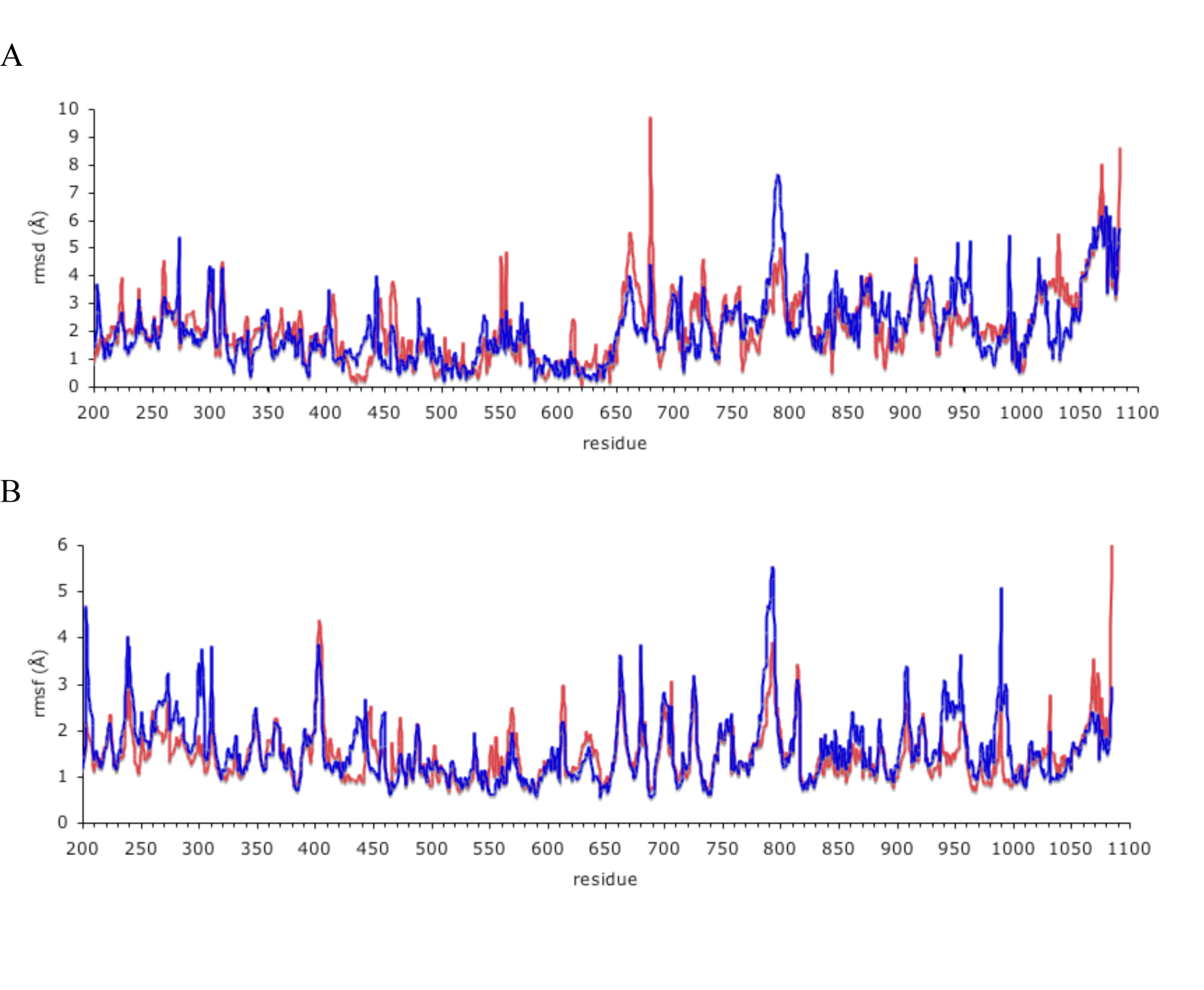

Supplement: Figure S3 — Per residue changes during the simulations. A. Per residue rms deviation relative to the starting structure after alignment using coordinates of the Cα atoms of domain II (392–649). Ligand-bound complex blue, apo red. B. Per residue rms fluctuations relative to the starting structure after alignment using coordinates of all Cα atoms. Ligand-bound complex blue, apo red. (TIF) [file pone.0028589.s003.tif]

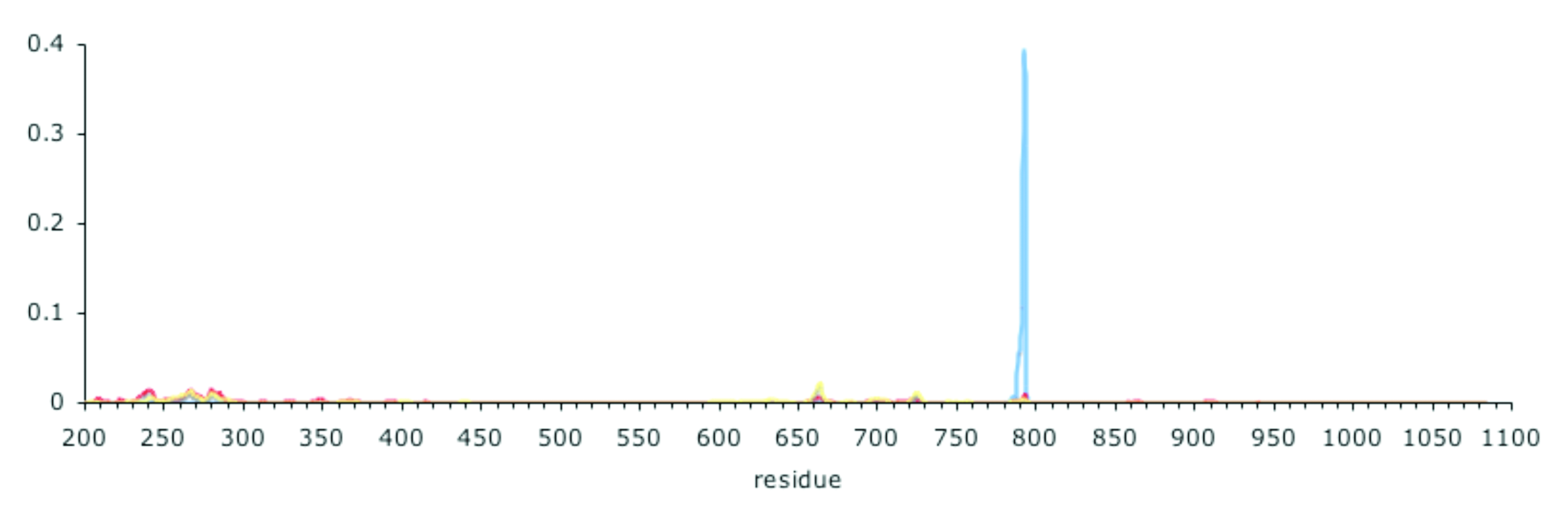

Supplement: Figure S4 — Elastic network analysis of Pf A-M1. Per residue fluctuations due to slowmodes 1–3 from the ANM analysis of PfA-M1 (PDB 3EBH). Units of fluctuations are relative only. Mode 1, blue; mode 2, red; mode 3, yellow. (TIF) [file pone.0028589.s004.tif]
