# Supplementary material for: The Plasmodium falciparum Malaria M1 Alanyl Aminopeptidase (PfA-M1): Insights of Catalytic Mechanism and Function from MD Simulations
Source: PLoS One. 2011 Dec 21;6(12):e28589. doi: 10.1371/journal.pone.0028589 (PMC3244404; doi:10.1371/journal.pone.0028589)
Supplement: Table S1 — This table shows the mean distances between the Zn apex atoms and the proximal coordinating atoms. (DOCX) [file pone.0028589.s005.docx]

**Table S1. Distances between the Zn apex atoms and the proximal coordinating atoms**^a^

|  | E519 oε1 | H496 nε2 | H500 nε2 | Water 1 O^b^ | Water 2 O^c^ |
| --- | --- | --- | --- | --- | --- |
| Apo^d^ | 1.19 ± 0.06 | 1.20 ± 0.04 | 1.19 ± 0.07 | 1.23 ± 0.07 | 2.07 ± 0.19 |
| Ligand^d^ | 1.19 ± 0.07 | 1.20 ± 0.04 | 1.21 ± 0.05 | 1.56 ± 0.32 | 1.53 ± 0.30 |

^a^ Mean values and standard deviations are in Å.

^b^ Corresponds to the position of the nucleophilic water (Fig.1C)

^c^ In the apo simulation, corresponds to the position of the second water oxygen, in the ligand-bound complex, corresponds to the position of the carbonyl oxygen.

^d^ From PfA-M1 simulations, distances sampled every 50ps over the 75ns simulations.
